# Genetic testing in intellectual disability psychiatry: Opinions and practices of UK child and intellectual disability psychiatrists

**DOI:** 10.1111/jar.12391

**Published:** 2017-08-23

**Authors:** Kate Wolfe, Kerstin Stueber, Andrew McQuillin, Fatima Jichi, Christine Patch, Frances Flinter, André Strydom, Nick Bass

**Affiliations:** ^1^ Division of Psychiatry University College London London UK; ^2^ Biostatistics Group Joint Research Office University College London London UK; ^3^ Guy's and St Thomas’ NHS Foundation Trust London UK

**Keywords:** clinical management, copy number variant, diagnosis, learning disability, service provision

## Abstract

**Background:**

An increasing number of genetic causes of intellectual disabilities (ID) are identifiable by clinical genetic testing, offering the prospect of bespoke patient management. However, little is known about the practices of psychiatrists and their views on genetic testing.

**Method:**

We undertook an online survey of 215 psychiatrists, who were contacted via the Royal College of Psychiatrist's Child and Adolescent and Intellectual Disability Psychiatry mailing lists.

**Results:**

In comparison with child and adolescent psychiatrists, intellectual disability psychiatrists ordered more genetic tests, referred more patients to genetic services, and were overall more confident in the genetic testing process. Respondents tended to agree that genetic diagnoses can help patient management; however, management changes were infrequently found in clinical practice.

**Conclusions:**

Differences are apparent in the existing views and practices of child and adolescent and intellectual disability psychiatrists. Developing training and collaboration with colleagues working in genetic services could help to reduce discrepancies and improve clinical practice.

## 1 INTRODUCTION

Approximately 1% of the population has a diagnosis of intellectual disability (ID), which is characterized by impairments in both intellectual and adaptive functioning and has its origin in the developmental period. Intellectual disability is an aetiologically heterogeneous disorder, with both environmental and genetic causes. Rapid advances in genomics have resulted in many new genetic causes of intellectual disability being delineated (Gilissen et al., 2014). Perhaps the better recognized genetic causes of intellectual disability occur when individuals exhibit a constellation of symptoms indicative of a known syndrome. In such instances, a specific genetic test may be indicated, for example single gene testing in Fragile X syndrome. However, where the individual's presentation is not clearly suggestive of a specific syndrome, chromosomal microarray analysis (CMA) is now typically considered the first line genetic investigation (Miller et al., 2010).

CMA can identify small losses and gains of genetic material. These losses (deletions) and gains (duplications) are referred to as copy number variations (CNVs). Loss or gain of genetic material can, in some instances, alter gene function and effect neurodevelopment. Several recurrent CNVs are associated with elevated risk for intellectual disability, as well as other co‐morbid phenotypes such as schizophrenia and epilepsy. For example the 22q11.2 deletion syndrome is associated with intellectual disability, but is also is one of the strongest risk factors for psychosis (Schneider et al., 2014).

Individuals with intellectual disability face obstacles accessing both physical and mental health services and health inequalities have been described (Emerson, Baines, Allerton, & Welch, 2012). Understanding the genetic aetiology of intellectual disability could help to address some of these inequalities by facilitating individualized care plans. For example, there are clinical management guidelines available for the 22q11.2 deletion syndrome. Screening for specific physical health conditions, including cardiac, renal and immunology investigations, and a comprehensive mental health assessment are recommended (Habel et al., 2014). Such guidelines offer good prospects for early intervention and optimized health care, although they are not yet available for every genetic cause of intellectual disability.

In the UK, infants and children presenting with developmental delay are often seen by paediatricians, who can initiate genetic investigations and where appropriate refer onto specialist child and adolescent mental health services (CAMHS). Adult services are generally provided by specialist intellectual disability psychiatrists. Referrals for genetic testing can be made to National Health Service (NHS) Regional Genetics Centres (RGCs), which offer clinical genetics expertise in syndromes, cascade testing and counselling. Inequities in access to genetic testing have, however, encouraged the mainstreaming of genetic investigations, with an increased emphasis placed on medical specialists ordering tests directly (Burton, 2011).

Intellectual disability is often associated with co‐morbid psychiatric disorders and/or behavioural problems. Recent estimates from United Kingdom (UK) primary care records show that approximately 21% of individuals with intellectual disability have a psychiatric disorder, 25% have some record of challenging behaviour, and 49% had been prescribed psychotropic drugs (Sheehan et al., 2015). Given that investigation of the cause of intellectual disability predominately occurs at diagnosis in childhood, there is a large cohort of adults, many with later onset psychiatric disorders, who have not had a diagnostic assessment utilizing the latest genetic technologies (Baker, Raymond, & Bass, 2012). We recently recruited 202 adults with idiopathic intellectual disability from UK psychiatry services and found that 11% had undiagnosed clinically relevant CNVs (Wolfe et al., 2017).

Whilst the role of specialist clinicians in ordering/referring for genetic testing is evolving, little is known about their current views and practices. We aimed to explore the attitudes and practices of UK psychiatrists working in CAMHS and adult intellectual disability psychiatry services on genetic testing in intellectual disability.

## 2 METHOD

Psychiatrists from UK CAMHS and adult intellectual disability psychiatry services were surveyed as to their attitudes towards and current use of genetic investigations using an online survey.

### 2.1 Survey

The questions were developed through consultation with intellectual disability psychiatrists, a clinical geneticist, a genetic counsellor, a genetic researcher and a statistician. Following a pilot, a number of the questions were amended, and the opportunity for open text responses was enabled. The 28‐item self‐administered survey was composed of yes/no responses, multiple choice Likert scale questions, numeric outcomes and free text responses (available in the Appendix). The survey was programmed not to force answers to questions and enable completion of the survey with missing responses. It was administered via the online service tool Survey Monkey (SurveyMonkey Inc. Palo Alto, California, USA).

### 2.2 Participants

The survey was distributed to members of the Faculty of Child and Adolescent Psychiatry and members of the Faculty of Psychiatry of Intellectual Disability via the Royal College of Psychiatrists’ mailing list. Psychiatrists were invited by email to participate in the survey. A participation reminder was sent after 1 week. Respondents were removed from the analysis if they were junior trainees or listed professions other than CAMHS psychiatry and adult intellectual disability psychiatry, if they lived outside the UK and if they had not seen any patients with intellectual disability in the previous 12 months.

### 2.3 Statistical analysis

Quantitative statistical analyses were undertaken using IBM SPSS Statistics for Windows, Version 22.0 (IBM Corp, Armonk, NY, USA). The analysis compared CAMHS psychiatrists (referred to henceforth as child psychiatrists) and adult intellectual disability psychiatrists (referred to henceforth as intellectual disability psychiatrists). Continuous outcome variables were analysed, using a *t* test where the data were normally distributed, and Mann‐Whitney U test for non‐normally distributed data. The chi‐squared test was utilized to test categorical outcome variables. Binary logistic regression was undertaken to test univariable factors related to ordering a genetic test. For Likert scale responses, the data were collapsed from 5 to 3 scale responses by merging “strongly agree” with “agree” and “strongly disagree” with “disagree” or “very frequently” with “frequently” and “very rarely” with “rarely.” To compare clinical confidence ratings, a composite confidence score was generated by assigning the 5‐point Likert scale responses a confidence value ranging from 1 for strongly disagree to 5 for strongly agree. These scores were then summed across the eight confidence measures to obtain an overall composite confidence score. Where analyses have been undertaken on a subset of the data set due to missing values the number of respondents in the analysis has been indicated. Significance has been set at 0.006 to account for multiple testing.

### 2.4 Thematic analysis

Open text responses were thematically coded using Nvivo qualitative data analysis software (QSR International Pty Ltd. Version 10, 2012). Three open text questions were included in the survey, focusing on the benefits and concerns of genetic testing in clinical practice. For the word cloud, all open text responses were included, and a word frequency analysis was undertaken using Nvivo. Word stemming was undertaken to combine variations of words from the same root (e.g., genetic and genetics). All words mentioned greater than 5 times, excluding common words, were inputted into Wordle^™^ (http://www.wordle.net/) for the creation of the word cloud.

## 3 RESULTS

Responses were received from 215 psychiatrists, comprising 121 child psychiatrists (56%) and 94 intellectual disability psychiatrists (44%); 56% were females (*n* = 121). The majority of respondents worked in England (*n* = 170, 80%), followed by Scotland (*n* = 29, 14%), Wales (*n* = 9, 4%) and Northern Ireland (*n* = 5, 2%). The majority of respondents worked in community teams (*n* = 115, 57%) followed by specialist assessment inpatient units (*n* = 23, 11%) and specialist referral centres (outpatient) (*n* = 19, 9%). A further 46 respondents (23%) reported that they worked in more than one of these settings. The median number of years working in the speciality was 10 (child psychiatrists 10 years, intellectual disability psychiatrists 11 years).

### 3.1 Attitudes towards genetic testing

Respondents were asked to estimate the percentage of people with intellectual disability for whom genetic factors make a significant contribution towards the cause of their intellectual disability. Estimates from child psychiatrists (Mean = 42%, *SD* = 24.7, Range = 2‐100%) were comparable to those of intellectual disability psychiatrists (Mean = 39.6%, *SD* = 23.1, Range=3‐90%) (*n* = 206, Mean difference = 2.4, 95% CI (−4.25, 8.1) *p* = .48). However, estimates by both child and intellectual disability psychiatrists of the percentage of patients on their caseloads with an established genetic diagnosis were much lower. Intellectual disability psychiatrists estimated a higher percentage of their own patients to have an established genetic diagnosis (Median = 10%, Range = 0‐70%) compared to child psychiatrists (Median=5%, Range = 0‐100%), (*n* = 205, *U* = 3661.5, Mean rank *= *120 versus Mean rank = 90, *p* = <.001).

### 3.2 Ordering of genetic tests

More intellectual disability psychiatrists (77%), compared with child psychiatrists (56%), had ordered a genetic test in the last 10 years (*n* = 162, χ² = 8.08, *p* = .004). Respondent's estimates of the percentage of intellectual disability caused by genetic factors did not influence the likelihood of them ordering a genetic test (*n* = 157, OR 1.01, 95% CI (0.99‐1.03), *p* = .19). The percentage of patients on respondents’ caseloads with an established genetic diagnosis also did not affect the likelihood of ordering a genetic test (*n* = 156, OR 1.02, 95% CI (0.99‐1.05), *p* = .33).

### 3.3 Confidence in the genetic testing process

Respondents were asked how confident they felt in eight aspects of the genetic testing process, as presented in Table 1. Child psychiatrists had a lower average total confidence score (Mean = 22.1, *SD* = 6.8) in comparison with intellectual disability psychiatrists (Mean = 27.4, *SD*=5.5). (*n* = 186, Mean difference = 5.3, 95% CI (3.42, 7.1), *p *= <.001). In comparison with child psychiatrists, intellectual disability psychiatrists agreed that they were confident in: knowledge of genetic tests (69% versus 29%); assessing for dysmorphic features (63% versus 47%); ordering (47% versus 24%) and interpreting genetic tests (35% versus 12%); genetic counselling (22% versus 12%) and feeding back test results to patients (64% versus 32%) and their families (68% versus 34%).

**Table 1:** Self rated confidence scores of child and intellectual disability psychiatrists (*n* = 186) in eight areas of the genetic testing process

	Psychiatry specialism	Disagree	Neither agree nor disagree	Agree
Knowledge of genetic tests	Child	52 (50%)	23 (22%)	30 (29%)
	Intellectual disability	11 (14%)	14 (17%)	56 (69%)
Assessing for dysmorphic features	Child	34 (32%)	22 (21%)	49 (47%)
	Intellectual disability	14 (17%)	16 (20%)	51 (63%)
Assessment of capacity to consent	Child	10 (10%)	12 (11%)	83 (79%)
	Intellectual disability	2 (3%)	6 (7%)	73 (90%)
Ordering genetic tests	Child	55 (52%)	25 (24%)	25 (24%)
	Intellectual disability	16 (20%)	27 (33%)	38 (47%)
Interpreting genetic test results	Child	70 (67%)	22 (21%)	13 (12%)
	Intellectual disability	31 (38%)	22 (27%)	28 (35%)
Feedback to patients	Child	43 (41%)	28 (27%)	34 (32%)
	Intellectual disability	13 (16%)	16 (20%)	52 (64%)
Feedback to family/carers	Child	41 (39%)	28 (27%)	36 (34%)
	Intellectual disability	14 (17%)	12 (15%)	55 (68%)
Genetic counselling	Child	71 (68%)	21 (20%)	13 (12%)
	Intellectual disability	36 (44%)	27 (33%)	18 (22%)

### 3.4 Concerns with the genetic testing process

Respondents were asked what their main concerns were in relation to the genetic testing process, see Table 2. Both child and intellectual disability psychiatrists agreed that lack of available treatment was one of the main concerns (58% versus 51% retrospectively). Another main concern was lack of resources, 54% of child and intellectual disability psychiatrists agreed that this was a concern. Implications for insurance were a bigger concern for child psychiatrists in comparison with intellectual disability psychiatrists (50% versus 38%), whereas issues around counselling were a bigger concern for intellectual disability psychiatrists (53% versus 43%).

**Table 2:** Concerns child and intellectual disability psychiatrists (*n* = 195) report in ten areas of the genetic testing process

	Psychiatry specialism	Disagree	Neither agree nor disagree	Agree
Stigma of patients/families	Child	42 (39%)	39 (36%)	27 (25%)
	Intellectual disability	39 (45%)	21 (24%)	27 (31%)
Lack of available treatment	Child	26 (24%)	19 (18%)	62 (58%)
	Intellectual disability	27 (31%)	16 (18%)	44 (51%)
Lack of resources	Child	23 (22%)	26 (24%)	58 (54%)
	Intellectual disability	21 (24%)	19 (22%)	46 (54%)
Implications for insurance	Child	23 (22%)	30 (28%)	53 (50%)
	Intellectual disability	30 (35%)	24 (28%)	33 (38%)
Misuse of results	Child	22 (21%)	39 (37%)	45 (43%)
	Intellectual disability	37 (43%)	17 (20%)	32 (37%)
Difficulty obtaining a family history	Child	40 (37%)	39 (36%)	28 (26%)
	Intellectual disability	27 (31%)	19 (22%)	41 (47%)
Obtaining a sample	Child	38 (36%)	37 (71%)	31 (29%)
	Intellectual disability	35 (40%)	23 (26%)	29 (33%)
Issues around counselling	Child	37 (35%)	24 (22%)	46 (43%)
	Intellectual disability	22 (25%)	19 (22%)	46 (53%)
Issues around capacity to consent	Child	31 (29%)	32 (30%)	44 (41%)
	Intellectual disability	36 (41%)	13 (15%)	38 (44%)

### 3.5 Feedback of results and clinical management

**Figure 1:**
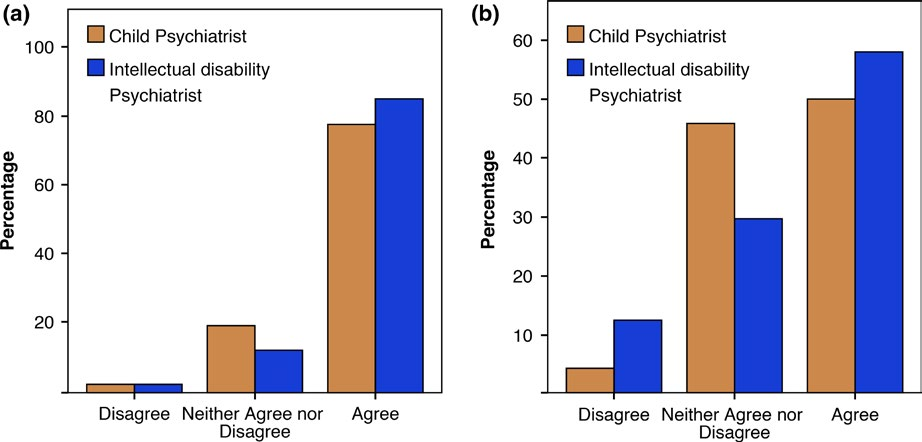
Percentage of child (*n* = 72) and intellectual disability psychiatrists (*n* = 81) who feel that a genetic diagnosis is helpful for family members (a) and patients with intellectual disability (b). [Colour figure can be viewed at wileyonlinelibrary.com].

As seen in Figure 1 both child and intellectual disability psychiatrists agreed that a genetic diagnosis is more beneficial for family members than patients. In comparison with child psychiatrists, intellectual disability psychiatrists were more inclined to agree that a diagnosis is beneficial for family members (85% versus 78%) (Figure 1a) and patients (58% versus 50%) (Figure 1b).

Respondents were also asked how they fed back results to their patients with intellectual disability. Of the 146 respondents eight (5%) had utilized videos, 20 (14%) had received input from speech and language therapists, 48 (33%) had used easy read materials, and 98 (67%) had used none of these aids. Responses were comparable for child and intellectual disability psychiatrists.

**Figure 2:**
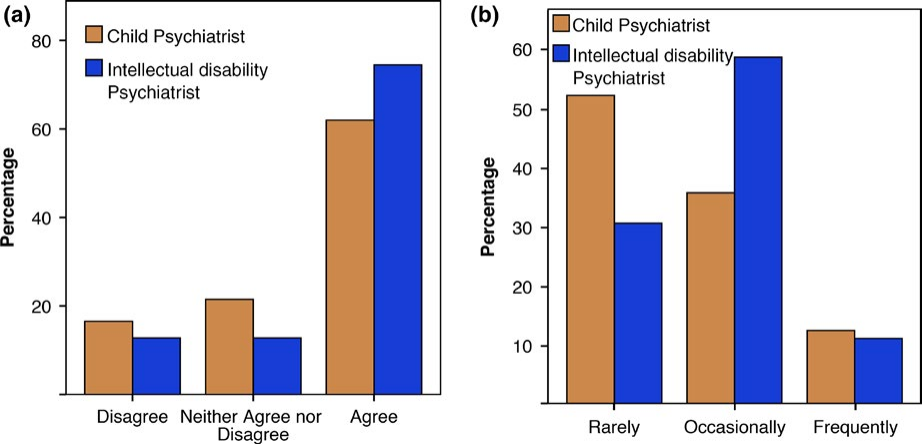
Percentage of child (*n* = 121) and intellectual disability (*n* = 94) psychiatrists who feel that that a genetic diagnosis is helpful for patient management (a) and who report that genetic information has helped their patient management (b) (child *n* = 73, intellectual disability *n* = 82). [Colour figure can be viewed at wileyonlinelibrary.com].

Figure 2 shows respondents’ views and experiences of clinical management changes following genetic diagnoses. Respondents agreed that a genetic diagnosis would help with patient clinical management (75% intellectual disability versus 62% child) (Figure 2a); however, few agreed that they had seen frequent management changes in their patients (11% intellectual disability versus 12% child) (Figure 2b).

### 3.6 Referral to genetics services

Respondents were asked if they had ever ordered a genetic test or made a referral to a clinical genetics service. Those who had made a referral were also asked to estimate the number of referrals in last year. A significantly higher percentage of intellectual disability psychiatrists, compared with child psychiatrists, had ordered a test or made a referral (90% versus 68%, *n* = 214, χ² = 15.92, *p* = <.001). Intellectual disability psychiatrists also referred more patients per year to the genetics clinic compared with child psychiatrists (*n* = 153, Range = intellectual disability 0‐25, child 0‐10, *U* = 2161.5, Mean rank = 87 versus Mean rank = 67, *p* = .004).

Respondents were asked what the main reasons for referral to clinical genetics services were. Of the 155 respondents, the most frequent reason for referral was presence of dysmorphic features (46% child, 57% intellectual disability) followed by intellectual disabilities (31% child, 38% intellectual disability). The least likely reason for referral was pharmacological treatment (2% both child and intellectual disability).

### 3.7 Service structure and training

Both intellectual disability and child psychiatrists agreed that closer links with regional genetics services would be helpful (83% versus 72%, *n* = 197). Respondents were also in agreement that they would prefer to refer to a regional genetics service rather than order a genetic test themselves (child 85%, 77% intellectual disability *n* = 195). Finally, there was a consensus that further training in genetics would be beneficial (child 71%, 66% intellectual disability, *n* = 195).

### 3.8 Thematic analysis

Four main themes were identified from the 76 respondents who completed the open response questions, comprising: family impact, clinical management, access to services, and training.

Of the 23 respondents who reported family impact the most frequent benefits identified were; relief from guilt and increased understanding of the patient's condition, followed by ability to access a support group and family planning. Respondents who discussed clinical management tended to mention the positive aspects, such as tailored medical and psychiatric interventions and clarification of syndrome‐specific behaviours. Only three respondents stated that they did not think a genetic diagnosis was helpful for clinical management. One respondent commented “*it is something of a paradox that the advances in the understanding of genetics and its potential impact upon our patient group has not translated into a significant increase in the use of genetic testing to help with diagnosis and care planning. I can only surmise that the social model of Disability as outlined in Valuing People has steered the diagnostic process away from genetic labelling*.”

Access to genetics services was mentioned by 22 respondents, who described problems with referring to genetics services and the variable levels of knowledge of professionals involved in the pathway. There was concern that psychiatrists, who have not specialized in genetics, do not have the skills to refer directly for genetic testing. Good working relationships with genetics services were said to be a valuable resource. Five child psychiatrists stated that they would defer to their paediatric colleagues to make decisions about genetic testing.

**Figure 3:**
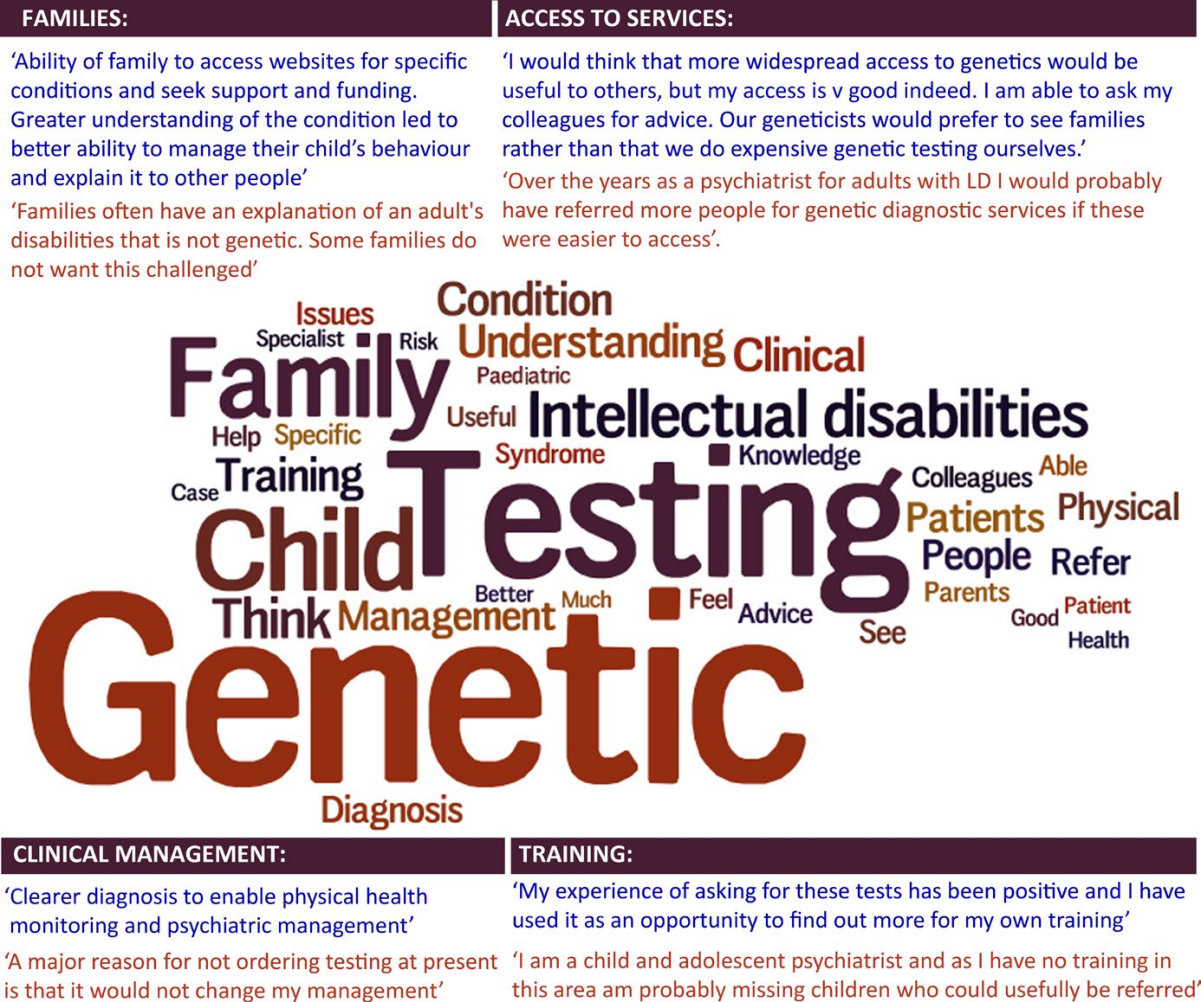
Word cloud of words mentioned 5 or more times from open text responses with larger words mentioned more frequently. Positive and negative responses from the main themes are displayed in the text boxes. [Colour figure can be viewed at wileyonlinelibrary.com].

Several respondents felt that current training in genetics was insufficient and is not keeping abreast of technological advances. It was suggested that quick reference guides and screening tools would be valuable resources to support the decision‐making process. See Figure 3 for a word cloud of the most frequently used words in the open text responses (results from both professional groups as responses were comparable for child and intellectual disability psychiatrists) and a summary of positive and negative opinions for each of the main themes.

## 4 DISCUSSION

Our results indicate that the majority of child and intellectual disability psychiatrists working with patients with intellectual disability are already ordering genetic tests or making referrals to genetics services. However, there are several disparities in clinical genetic practices. In comparison with child psychiatrists, intellectual disability psychiatrists reported the following: a higher number of patients with genetic diagnoses, greater confidence in the genetic testing process, higher numbers of tests ordered and more patients referred per year to genetics services.

Respondents were asked to estimate the percentage of intellectual disability caused by genetic factors. The responses varied greatly with some respondents estimating as low as 2% and others as high as 100%. Although both child and intellectual disability psychiatrists had similar mean estimates (39.6% and 42%) of the percentage of intellectual disability caused by genetic factors, these estimates were much higher than the actual percentage of patients on caseloads with a known genetic diagnosis (median = 10% intellectual disability and median=5% child). It is unclear why this disparity exists. It would be interesting to investigate whether the clinician responsible for ordering genetic testing is communicating the results to other professionals involved in the individual's care. This will be particularly important for individuals with intellectual disability and co‐morbid diagnoses who are under the care of multiple medical professionals.

A high proportion of intellectual disability psychiatrists (77%) and just over half of child psychiatrists had directly ordered a genetic test. In comparison with child psychiatrists, intellectual disability psychiatrists were significantly more likely to order a genetic test and also referred more patients to the genetics clinic per year. This may have in part been a reflection of the intellectual disability psychiatrist's greater reported confidence in the genetic testing process. As evidenced in Table 1, intellectual disability psychiatrists were significantly more confident in all aspects of the testing process, apart from capacity testing which is likely to be more complex in adulthood. One explanation for intellectual disability psychiatrists being more confident and ordering/referring for more genetic tests is due to the different structures of child and adult intellectual disability psychiatry services. Some child psychiatrists reported that they would defer to paediatric colleagues for opinions on genetic testing.

The survey highlighted a number of barriers to genetic testing in clinical intellectual disability services. Both child and intellectual disability psychiatrists reported that they were concerned about lack of available treatment and resources for genetic testing. Interestingly child psychiatrists had specific concerns about implications for insurance. The Department of Health has released a moratorium extending until 2019, whereby the only genetic test required to be disclosed is for Huntington's disease on life insurance sums worth more than £500,000 (HMGovernment, 2014). Therefore, results from CMA should have no impact on insurance premiums, and this misconception could be a barrier to clinicians ordering/referring for genetic testing. Intellectual disability psychiatrists expressed concern about issues surrounding counselling. Feedback of genetic diagnoses to adults with intellectual disability is understandably more complex than feedback to parents of children with intellectual disability, and this could be an important area for additional resources and research.

Both child (85%) and intellectual disability (77%) psychiatrists agreed that they would prefer to refer to a Regional Genetics Centre (RGC) rather than directly order a genetic test themselves; however, links with NHS RGCs appeared to be variable. Some respondents reported good links with their local genetics services, whilst others felt that access to services was a barrier to referring for genetic testing. Both intellectual disability (83%) and child psychiatrists (72%) felt that better links with genetics services would be beneficial. Many of these clinicians felt that they do not have the knowledge or training to order genetic tests directly. This finding is supported by another survey, which found intellectual disability psychiatrists lacked adequate knowledge about genetics and testing processes (de Villiers & Porteous, 2012).

The majority of respondents expressed a wish for further training (71% child, 66% intellectual disability). Neither child and adolescent nor intellectual disability psychiatry curricula currently have learning objectives that specifically cover genetic disorders associated with intellectual disability (http://www.rcpsych.ac.uk/traininpsychiatry/corespecialtytraining/curricula.aspx). The curricula also fail to cover the genetic work‐up and basic genetic counselling skills that are required to take more of an active role in identifying and managing patients with genetic disorders. However, there are several recent initiatives to improve the psychiatry curriculum. For example, the Gatsby‐Wellcome initiative aimed to ensure that training focuses more on scientific advances in basic and clinical neurosciences (http://www.rcpsych.ac.uk/traininpsychiatry/corespecialtytraining/neuroscienceproject.aspx). It is, therefore, hoped that future cohorts of psychiatrists will be more confident in utilizing technological advancements in the assessment and management of their patients.

One of the reasons for undertaking genetic investigations is that a genetic diagnosis is likely to provide information about specific associated medical and psychiatric phenotypes and thus could improve treatment plans and clinical management for the patient. Whilst the majority of respondents felt that a genetic diagnosis would help with clinical management, fewer patients on their caseloads had a genetic diagnosis than they would expect, and clinical management changes following genetic diagnoses were not frequently seen in practice. There are published medical guidelines available for several genetic disorders, for example via the Orphanet portal for rare diseases (http://www.orpha.net/), and information guides on an extensive range of chromosomal disorders are available from the support group Unique (http://www.rarechromo.co.uk/html/DisorderGuides.asp). It would be of interest for further research to investigate whether psychiatrists are aware of these guidelines when they receive a genetic diagnosis for their patient.

Another important consideration is that knowledge of behavioural phenotypes can place psychiatrists in a better position to deliver appropriate interventions and environmental adaptations. Whilst there is within syndrome variation it has been shown that certain behavioural features, such as repetitive and self‐injurious behaviours, are more common in particular syndromes. There are also implications for health screening, for example gastro‐intestinal problems are common in Cornelia de Lange syndrome and can exasperate self‐injurious behaviours (Waite et al., 2014). A recent survey of intellectual disability professionals found that nine out of ten professionals interviewed felt that specific knowledge of a neurodevelopmental syndrome should play a key role in healthcare provision. A specific genetic diagnosis was thought to prompt proactive screening for related physical and mental health problems, which is of particular benefit for patients with severe impairments (Redley, Pannebakker, & Holland, 2016). One of the main challenges in practice is that individual syndromes are rare and psychiatrists are unlikely to care for many individuals with the same disorder, although the overall burden of rare syndromic disorders is large.

Both child and intellectual disability psychiatrists agreed that receiving a genetic diagnosis was more beneficial for family members than for the patient. Research has shown that there is a benefit to mothers in receiving a diagnosis for a child with intellectual disability; however, there is a lack of research as to the impact of a genetic diagnosis for adults with intellectual disability (Lingen et al., 2016) Several respondents reported that a diagnosis can help to alleviate guilt for family members, as well as increasing understanding of the patient's syndrome‐specific behaviours and enabling valuable access to support groups. It seems that respondents were able to report on a range of psychosocial benefits, which could indirectly improve patient management; however, tangible changes in clinical decision making following a genetic diagnosis were less easy to define.

### 4.1 Limitations

The survey was self‐reported which could have led to biases in estimations. There may have been a selection bias in the clinicians who chose to respond to the survey, perhaps those with more extreme views on genetics were more inclined to respond. This survey specifically focused on psychiatrists, who are one of the medical specialists frequently in contact with patients with intellectual disability in the UK. These findings may not be generalisable to other countries where services are organized differently.

## 5 CONCLUSIONS

Whilst a high number of child and intellectual disability psychiatrists appear to already be ordering genetic tests there remains a preference for referring directly to clinical genetics services. Respondents highlighted several areas of the genetic testing process in which they particularly lack confidence, such as indications for testing, interpretation and feedback of genetic results. Child psychiatrists in particular felt less confident, ordered fewer genetic tests, and referred fewer patients to genetic services.

Genetic investigations are continuing to advance at a very rapid pace, with exome and whole‐genome sequencing beginning to enter clinical practice. In conjunction with other genetic investigations, it is likely that a genetic diagnosis will be identifiable in a much higher proportion of patients with intellectual disability in the future. This should facilitate early diagnosis and tailored interventions for patients and their families. However, as the landscape of genetic investigations becomes more complex it is going to be a challenge for psychiatrists to keep pace of developments. Improvements in training and closer links with genetics services would appear to be key areas to address to meet this challenge.

## CONFLICT OF INTEREST

There are no conflicts of interest.

## APPENDIX 1 SURVEY ON ATTITUDES OF CLINICIANS TOWARDS GENETIC TESTING IN PEOPLE WITH INTELLECTUAL DISABILITIES

### 1.1

*1) What is your profession?*

**Table:**

□ Doctor working in psychiatry of intellectual disability
□ Doctor working in child and adolescent psychiatry
□ Other please specify:______________________________________________________

*2) Where do you work?*

**Table:**

□ Scotland
□ England
□ Northern Ireland
□ Wales
□ Other please specify:______________________________________________________

*3) What is your gender?*

**Table:**

□ Female
□ Male
□ I prefer not to state

*4) In what setting do you work?*

**Table:**

□ Community team
□ Specialist referral centre (outpatient)
□ Specialist referral centre (inpatient)
□ Other please specify:______________________________________________________

*5) Years working in your specialty?*

____years

*6) How many patients with developmental delay/ID have you seen in the last 12 months in the following categories approximately (please state a number for each category)?*

- Number of children (0‐12 years):__________
- Number of adolescents (>12‐18 years): __________
- Number of adults (>18 years): __________

*7) In general do you think **making a genetic diagnosis** would help with management of your patients?*

**Table:**

**Strongly agree**	**Agree**	**Neither agree nor disagree**	**Disagree**	**Strongly disagree**
□	□	□	□	□

*8) I estimate that there is a significant genetic contribution towards the cause of the ID in …. % people with ID*:

- ______________% of people with ID.

*9) I estimate that a significant genetic contribution towards the cause of the ID has been identified in…. % of my patients*:

- ______________% of my patients.

*10) Have you ever ordered a genetic test/made a referral to a geneticist in a patient with an intellectual disability?*

**Table:**

□ Yes
□ No‐ **please continue with question 21**

*11) Please estimate how many patients with ID you have referred to a genetics clinic in the last year (please state a number)?*

- ______________(number of patients).

*12) Reasons for genetic testing/referral to genetics?*

**Table:**

	Very frequently	Frequently	Occasionally	Rarely	Very rarely
Intellectual disability:	□	□	□	□	□
Psychiatric disorder:	□	□	□	□	□
Medical conditions:	□	□	□	□	□
Prediction of risk for	□	□	□	□	□
Inheritance of family member:					
Family planning of the patient:	□	□	□	□	□
Dysmorphic Features:	□	□	□	□	□
Pharmacological treatment:	□	□	□	□	□
Other:	□	□	□	□	□
Other (please specify):_______________________________________________________________					

*13) Have you directly ordered a genetic test in a patient with ID in the last 10 years?*

**Table:**

□ Yes
□ No‐ **please continue with question 17**

*14) Please estimate how many genetic tests have you ordered directly in patients with ID/developmental delay in the last 12 months/last 10 years approximately (please state a number)*:

- Last 12 months (number of tests):__________
- Last 10 years (number of tests): __________

*15) Please indicate which of the following tests you have ordered in patients with ID (please tick all that apply)?*

**Table:**

□ Karyotype analysis
□ MLPA
□ FISH
□ Single gene mutation detection
□ Array CGH
□ Other, please state: ___________________________________________________________

16) *Please indicate your first line genetic investigations in patients with ID/developmental delay (please tick all that apply)?*

**Table:**

□ Karyotype analysis
□ MLPA
□ FISH
□ Single gene mutation detection
□ Array CGH
□ Not applicable
□ Other, please state: ___________________________________________________________

*17) Has the genetic information changed your management?*

**Table:**

Very frequently	Frequently	Occasionally	Rarely	Very rarely
□	□	□	□	□

*18) I have used the following to aid communication in genetic counselling (please tick all that apply)*:

**Table:**

□ Videos
□ Easy read material
□ Speech and Language Therapist
□ None of the above
□ Other, please state: ___________________________________________________________

*19) Generally I feel a genetic diagnosis has been helpful to the **patient** with ID*:

**Table:**

**Strongly disagree**	**Disagree**	**Neither agree nor disagree**	**Agree**	**Strongly agree**
□	□	□	□	□

*20) Generally I feel a genetic diagnosis has been helpful for the patient's **family***:

**Table:**

**Strongly disagree**	**nor disagree**	**Neither agree**	**Agree**	**Strongly agree**
□	□	□	□	□

*21) Thinking about the assessment process in CHILDREN/ADOLESCENTS with ID: I feel confident in (not applicable = N/A)*:

**Table:**

	**N/A**	**Strongly Disagree**	**Disagree**	**Neither Agree nor disagree**	**Agree**	**Strongly Agree**
Knowledge of indications/ genetic tests:	□	□	□	□	□	□
Assessing for dysmorphic features:	□	□	□	□	□	□
Genetic counselling:	□	□	□	□	□	□
Assessment of capacity to consent:	□	□	□	□	□	□
Ordering genetics tests:	□	□	□	□	□	□
Interpreting genetic test results:	□	□	□	□	□	□
Feedback to patients:	□	□	□	□	□	□
Feedback to family / carers:	□	□	□	□	□	□

*22) Thinking about the assessment process in ADULTS with ID: I feel confident in (not applicable = N/A)*:

**Table:**

	**N/A**	**Strongly Disagree**	**Disagree**	**Neither Agree nor disagree**	**Agree**	**Strongly Agree**
Knowledge of indications/ genetic tests:	□	□	□	□	□	□
Assessing for dysmorphic features:	□	□	□	□	□	□
Genetic counselling:	□	□	□	□	□	□
Assessment of capacity to consent:	□	□	□	□	□	□
Ordering geneticstests:	□	□	□	□	□	□
Interpreting genetictest results:	□	□	□	□	□	□
Feedback to patients:	□	□	□	□	□	□
Feedback to family / carers:	□	□	□	□	□	□

*23) I think closer links with the regional genetics service would be helpful*:

**Table:**

**Strongly agree**	**Agree**	**Neither agree nor disagree**	**Disagree**	**Strongly disagree**
□	□	□	□	□

*24) I prefer to refer to a genetics clinic (rather than ordering the tests myself)*:

**Table:**

**Strongly agree**	**Agree**	**Neither agree nor disagree**	**Disagree**	**Strongly disagree**
□	□	□	□	□

*25) I would like further training in clinical genetics*:

**Table:**

**Strongly agree**	**Agree**	**Neither agree nor disagree**	**Disagree**	**Strongly disagree**
□	□	□	□	□

*26) I am concerned about the following with respect to genetic testing in ID*:

**Table:**

	**Strongly Agree**	**Agree**	**Neither Agree nor disagree**	**Disagree**	**Strongly Disagree**
Stigma of patients/families:	□	□	□	□	□
Lack of available treatment:	□	□	□	□	□
Lack of resources:	□	□	□	□	□
Implications for insurance:	□	□	□	□	□
Misuse of results:	□	□	□	□	□
Difficulty of obtaining a family history:	□	□	□	□	□
Obtaining a sample:	□	□	□	□	□
Issues around counselling:	□	□	□	□	□
Issues around capacity to consent:	□	□	□	□	□
Other:	□	□	□	□	□
Other (please specify):_______________________________________________________________					

*27) Any other specific benefits that came out of genetic testing*:

*28) Any other suggestions/comments?*
